# Does Fintech affect the psychological traits of managers? Based on the perspective of manager overconfidence

**DOI:** 10.3389/fpsyg.2022.1008944

**Published:** 2022-12-09

**Authors:** Liang Wang, Wenyi Xiao, Dequan Huang

**Affiliations:** ^1^College of Business, Shanghai University of Finance and Economics, Shanghai, China; ^2^School of European Languages and Cultures, Beijing Foreign Studies University, Beijing, China; ^3^School of Finance, Guangdong University of Finance and Economics, Guangzhou, China

**Keywords:** psychological trait of manager, China, Fintech, manager overconfidence, power concentration, financing constraints

## Abstract

Overconfidence has undertaken an indispensable role in the psychology of managers and places important significance on managers’ behavior and decision-making. This study investigates the effect of Fintech on the psychological traits of managers from the perspective of overconfidence based on the panel data of Chinese A-share non-financial listed firms and the digital inclusive finance index of Chinese prefecture-level cities between 2011 and 2020. The empirical results show that (1) Fintech exerts a negative effect on manager overconfidence; (2) the main channels of the negative effect of Fintech on manager overconfidence include Fintech coverage breadth and Fintech usage depth; (3) for firms with severe financing constraints and lower power concentration, the negative effect of Fintech on manager overconfidence is more prominent; and (4) our benchmark results still hold after a series of robust tests, including IV regression, altering the measurement of Fintech and manager overconfidence, and employing logit model re-estimation. Based on the above findings, this study provides some insights into the cause for managers’ psychological traits, maintaining managers’ mental health, and empowering the firms’ sustainable development by adopting Fintech.

## 1 Introduction

Fintech logged rapid expansion in many countries, including China. As a representative of the digital economy, Fintech has developed rapidly in China. On one hand, China’s 14th 5-Year Plan and the outline of its 2015 Vision emphasize “the steady development of financial technology and the acceleration of the digital transformation of financial institutions.” On the other hand, the Fintech Development Plan (2022–2025) issued by the People’s Bank of China also points out that we should promote higher-quality development of Fintech. In the context of globalization of science and technology, Fintech is also applied acutely in firms and the finance industry. The development of Fintech has not only driven the development of some emerging industries but also replaced some simple and repetitive work in the traditional financial industry and firms. Therefore, to clarify the consequences caused by Fintech development which not only has certain practical significance in China but also provides insights into the development of Fintech in emerging market countries.

Fintech is a technology-driven financial innovation that relies on big data, artificial intelligence, cloud computing, blockchain, and other information technologies and has an important impact on traditional financial services (Beck et al., 2016; Gomber et al., 2017). The integration of technology and finance has brought new changes in the business model that are deeply superimposed on the development of financial markets and institutions and have optimized the resource allocation of the market (Gambacorta et al., 2022). The influence of Fintech on the environment and individuals cannot be ignored. The past 5 years have seen a fruitful debate on the economic consequence of Fintech (Goldstein et al., 2019; Thakor, 2020; Bollaert et al., 2021). For instance, Fintech is a technological innovation for financial services, enhancing information acquisition and credit screening in firms and financial sectors (Fuster et al., 2019). Fintech smooths the interactions between banks and firms, improves information symmetry, facilitates corporate financing (Sedunov, 2017; Yin et al., 2019), fosters a better information environment, and optimizes corporate investment efficiency (Lv and Xiong, 2022).

A vast of literature discusses how corporate information acquisition and information environments are enhanced by Fintech (Fuster et al., 2019; Murinde et al., 2022). It is evident that the development of Fintech is associated with corporate information acquisition and information environments, which have potentially caused some psychological consequences at the firm level. Therefore, in the context of the rapid development of Fintech, it is surprising that few studies focus on the psychological consequences of Fintech at the firm level. Moreover, there is still no clear conclusion on the impact of Fintech on managers’ overconfidence. The impact of Fintech on managers’ overconfidence remains unexplored as a critical aspect of entrepreneurs’ psychological problems. It is important to fully consider whether and how the development of Fintech affects manager overconfidence.

Manager overconfidence, defined as the overestimation of “one’s own abilities, performance, level of control, or chance of success” (Malmendier and Tate, 2005), is a particularly pertinent personality factor, as it is prevalent among executives. The literature extensively suggests that manager overconfidence plays a decisive role in corporate policy. Manager overconfidence is associated with unprofitable mergers and inefficient investment. Then, many psychologists present evidence that most people believe they have above-average driving skills (Alicke et al., 1995). Managers may be particularly susceptible to this bias because overconfidence is stronger among talents (Lin et al., 2005; Goel and Thakor, 2008; Bouzouitina et al., 2021; Li and Tong, 2022). Therefore, manager overconfidence is a popular topic in psychology research (Kyle and Wang, 1997; Gervais et al., 2011; Tenney et al., 2019).

A considerable amount of literature has been published on the consequence of manager overconfidence, ranging from individual to firm factors (Lin et al., 2005; Malmendier and Tate, 2005; Bouzouitina et al., 2021; Li and Tong, 2022). The effect of manager overconfidence has been demonstrated extensively, which implies the economic importance of manager overconfidence. However, little attention is paid to the role that Fintech plays in affecting manager overconfidence. We attempt to investigate the role that Fintech plays in affecting manager overconfidence. Specifically, can the development of financial technology reduce managers’ overconfidence? It is an interesting question that needs to be answered.

To further explore the relationship between Fintech and manager overconfidence, we hand-collect and match two databases containing Chinese A-share non-financial listed firms and the digital inclusive finance index of Chinese prefecture-level cities between 2011 and 2020. Then, we construct a regression model to investigate the effect of Fintech and manager overconfidence. The empirical results show that (1) Fintech has a negative effect on manager overconfidence which is robust to a series of robustness checks, including IV regression and alter the measurement of Fintech and manager overconfidence; (2) the main channels of the negative effect of Fintech on manager overconfidence include Fintech coverage breadth and Fintech usage depth; and (3) for firms with severe financing constraints and lower power concentration, the negative effect of Fintech on manager overconfidence is more obvious.

This study contributes to the Fintech and manager overconfidence literature in three ways by examining whether and how Fintech can influence manager overconfidence. First, this study links to the previous literature about the negative consequence of Fintech (Sedunov, 2017; Fuster et al., 2019; Goldstein et al., 2019), especially on manager overconfidence, which unravels the effect of Fintech in making managers becomes more rational from the perspective of manager overconfidence. Second, this directly speaks to studies on the influence factors of manager overconfidence (Tenney et al., 2019; Serra-Garcia and Gneezy, 2021; Sudzina et al., 2021). We add to this strand of literature by providing solid empirical evidence on the negative effects of Fintech, which serve a vital role in firms’ sustainable development. Third, this study provides reliable evidence, deepens the understanding of the benefit of Fintech, and provides insights into the alleviation force of manager overconfidence in the background of the emerging market (Ionescu, 2020; Lewandowska et al., 2021; Liu et al., 2021; Morales et al., 2022).

The remainder of this study is structured as follows: Section 2 “Literature review and hypothesis development” contains literature and hypothesis development. Section 3 “Empirical models, sample selection, and variables” describes the empirical strategy, including empirical models, sample selection, and variables. Section 4 “Results and discussion” provides benchmark results and discussion. Section 5 “Robust check” conducts a series of robustness checks. Section 6 “Heterogeneous analyses” focuses on heterogeneous analyses from the perspective of financing constraints and power concentration. Section 7 “Conclusion” provides the conclusion and insights.

## 2 Literature review and hypothesis development

Fintech is a technology-driven financial innovation that relies on big data, artificial intelligence, cloud computing, blockchain, and other information technologies and constructs a sustained and significant impact on traditional financial services (Beck et al., 2016; Gomber et al., 2017; Goldstein et al., 2019). The high integration of technology and finance has brought new business model reformations with a deep superposition for the development of financial markets and institutions and improved the resource allocation of the market (Stein, 2002), which contributes greatly to enhancing the capital investment of enterprises in the real economy. The existing literature mainly focuses on the research of Fintech on the development of the financial industry and corporate micro-behavior. From the perspective of the financial industry, according to the technology spillover effect, traditional financial institutions can cooperate with Fintech companies to identify high-quality customers by processing a large amount of information, which improves the efficiency of banks’ deposit and loan business (Beck et al., 2016). The rapid rise of Fintech reaches long-tail customers that are difficult for financial institutions to reach, which has impacted the debt scale of commercial banks to a large extent, meanwhile having a negative impact on the overall operating performance of banks (Schmidt and Druehl, 2008). From the perspective of firms, a growing strand of literature started to pay attention to the impact of financial technology on the micro-behavior of corporate and firm managers. According to the existing literature, there is no literature on the relationship between Fintech and manager overconfidence. Many scholars have studied the impact of financial technology on economic consequences, but few on the role of “financial technology–information asymmetry–overconfidence.” There is also a lack of mechanism for Fintech to affect managers’ overconfidence. This study attempts to explore its impact on managers’ overconfidence from the perspective of financial technology.

A growing number of studies focus on the impacts of Fintech in the context of emerging markets. Lewandowska et al. (2021) hold that financial support is important for investments as a lever for developing SME innovativeness in Poland. Liu et al. (2021) find that SME’s SCF adoption positively impacts its performance but negatively impacts its risk. In addition, Stankovic et al. (2021) propose a methodology for measuring digital competitiveness using a composite index approach including various indicators. Popova (2021) determines the economic basis for the projects implemented by the Fintech company and determines the source of the efficiency of these companies in financial operations compared to the conventional bank. The Fintech outcomes, such as mitigating uncertainty in emerging markets, also draw some scholars’ attention (Ionescu, 2020; Vasenska et al., 2021).

The corporate operating environment often exerts influence on psychological biases for corporate executives and breeds a tendency of overconfidence. Specifically, Malmendier and Tate (2005) found that, compared with rational managers, overconfident managers tended to overestimate the value of the firm’s future cash flow and believed that the current value of the firm’s securities was undervalued by the market, which in turn led to the firm facing high external financing costs. Moreover, Fintech has solved the financing dilemma of firms by reducing information asymmetry, broadening financing channels and speeding up loan approval speed, reducing the external financing cost of firms, and reducing the overconfidence of managers caused by high financing costs. In addition, it is assumed that overconfidence as a certain aspect of manager individual psychology can be adopted as a strategy or maintained by selection, in a period of uncertainty and low level of information (Goel and Thakor, 2008; Li and Tong, 2022).

Fintech has solved the financing dilemma of firms by reducing information asymmetry, broadening financing channels and speeding up loan approval, and reducing the level of overconfidence of managers. First, the information asymmetry between banks and firms greatly reduces the efficiency of resource allocation in the credit market (Gambacorta et al., 2022; Murinde et al., 2022). Traditional financial institutions mainly rely on hard information such as financial data, the number of mortgages, and the guarantees of enterprises to conduct risk assessments and make loan decisions (Stein, 2002; Fuster et al., 2019; Berger et al., 2022). The lack of collation of “soft information” of firms is an important reason for firms to bear high financing costs (Ding et al., 2018). However, modern financial innovation technology provides high-quality technical tools for banks to analyze all-round information of small- and medium-sized firms, enhances the information transparency of small- and medium-sized firms, and improves the capital shortage of high-quality firms, thus effectively inhibiting firms from holding high levels of demand for cash. Second, with the rapid rise of financial technology, various financial service systems emerged, such as Ant Financial, which have subverted the credit intermediary model of traditional banks and directly penetrated physical operations, thereby effectively alleviating the problem of short sellers in corporate financing channels (Gomber et al., 2017; Bouzouitina et al., 2021). Banks continue to add new financing tools to support the growth of small- and micro-firms. The loan business has expanded financing channels for small- and medium-sized firms. Third, for firms that do not meet the rigid conditions for bank loans, commercial banks have built a more complete credit evaluation model, risk prevention and control mechanism, and loan approval system with the help of financial technology, providing firms with “less process, faster lending” credit service, which is also a manifestation of the technological spillover effect of Fintech.

Simultaneously, Fintech companies such as Ant Group have used Internet financial technology to reduce the loan review business time of traditional Chinese commercial banks from up to several months to 3 s. The rapid big data processing technology empowered by Fintech brings down the marginal cost of lending (Goldstein et al., 2019), which can help firms obtain financial support faster, effectively build a bridge between supply and demand of inclusive finance, and boost the financing efficiency of firms, thereby improving the financing environment of firms and reducing the level of overconfidence of managers. In addition, with the rapid development of financial technology, commercial banks have strengthened their ability to identify high-quality firms with the help of financial technology tools, eased the degree of information asymmetry between banks and firms, and benefited to form a more complete credit evaluation system and further improve the quality of firms. Loans are appropriately priced, thereby reducing the external financing costs faced by the firms. Therefore, the rapid development of Fintech endues the decrease in the degree of overconfidence of managers due to the high cost of external financing. Based on the previous elaboration, it can be established that the integration of finance and technology improves the financing difficulties and information environment, thereby reducing the level of overconfidence of managers. Therefore, this study proposes the following hypothesis H1.

**H1:** The development of financial technology reduces the level of overconfidence of managers.

## 3 Empirical models, sample selection, and variables

### 3.1 Sample coverage and data sources

We combine two data sources to examine the effect of digital financial inclusion on manager overconfidence. The first data source is the China Stock Market Accounting Research (CSMAR) database, which contains detailed information about all Chinese listed firms’ top management team (TMT) and board members as well as annual reports and firm financial information. The second data source is the “The Peking University Digital Financial Inclusion Index of China” database, which is one of the most comprehensive databases of digital financial inclusion and provides detailed data on Chinese prefecture-level data of the digital financial inclusion index, including the index of the digital financial inclusion index and other important dimensions (such as coverage breadth, digitization level, and usage depth).

This study merges the above two databases by matching the firm-registered location (Prefecture-city level). Specifically, to test the relationship between Fintech and manager overconfidence, we match firm-specific variables (which include manager overconfidence and a set of control variables) with prefecture-city variables (which include a series of Fintech variables in the prefecture-city level) according to registered locations of firms. Due to the sample period of variables related to Fintech provided by “The Peking University Digital Financial Inclusion Index of China” limit in 2011–2020, we also chose all Chinese non-financial publicly listed companies listed on the Shanghai and Shenzhen exchanges between 2011 and 2020. The sample data structure belongs to panel data, which contains two dimensions (firm and year). Our main variables vary across firms and years simultaneously. Because we merged the above two databases by manually matching firms’ registered location addresses, measurements of Fintech also vary across the firm’s registered location and year simultaneously. In addition, the firms’ registered locations may change with the year.

On account of Chinese listed companies representing not only the driving force in Fintech innovation in China but also playing an important role in the development of Fintech in China (Guo and Liang, 2016), we select Chinese A-share non-financial listed firms as research samples of this study. We obtain our regression sample for the empirical analysis by merging the above two databases by manually matching firms’ registered location addresses. The raw sample includes 16,067 firm-year observations. Following previously related research (Malmendier and Tate, 2005; Fuster et al., 2019; Lyons et al., 2021), we impose the following restrictions: (1) Because the leverage of financial listed firm is abnormal, this study deletes firms belonging to the financial industry; (2) this study deletes observations with insufficient data for calculating financial and corporate governance variables; and (3) this study deletes firms that were specially treated. Our final sample includes 16,067 firm years. This study winsorizes the continuous variables at the 1 and 99% levels. Table 1 lists the definitions of all the variables, and Table 2 reports the summary of statistics.

**TABLE 1:** Variable definitions.

Dependent variables	Definitions
*OC*	Follow the measurement proposed by Lin et al. (2005)
**Explanatory variables**	
Fintech	The Peking University digital financial inclusion index of firm’s registered location
**Control variables**	
*STATE*	A dummy variable for private firms.
*SIZE*	Natural logarithm of total assets
*LEV*	Ratio of total liabilities divided by total assets
*AGE*	Number of years after the firm’s establishment
*Growth*	Tobin Q
*GP*	Operating profit ratio
*AS*	Ratio of liquid assets relative to total assets
*BOARD*	Natural logarithm of the number of firm’s board of directors
*SR*	The shareholder ratio of management team

**TABLE 2:** Descriptive statistics of the main variables.

Variable	*N*	Mean	Median	Standard deviation	Min	Max
*OC*	16,067	0.868	1	0.338	0	1
Fintech	16,067	1.921	2.010	0.655	0.231	3.030
*SR*	16,067	0.167	0.021	0.224	0	5.910
*LEV*	16,067	0.407	0.390	0.252	0.007	13.4
*Growth*	16,067	2.786	2.060	2.794	0.673	122.2
*GP*	16,067	0.296	0.266	0.178	–0.649	1
*AS*	16,067	0.583	0.599	0.202	0.009	0.999
*SIZE*	16,067	22	21.820	1.292	16.76	28.51
*BOARD*	16,067	2.131	2.197	0.199	1.099	2.890
*AGE*	16,067	2.690	2.773	0.416	0	3.932
*STATE*	16,067	0.307	0	0.461	0	1

### 3.2 Empirical models

To estimate the effects of Fintech on manager overconfidence, we use a regression model to estimate the effect. We construct the regression model as follows:


(1) OC=i⁢tα+βF1intech+i⁢tγ⋅CVs+i⁢tμ+iθ+tei⁢t

where i and t indicate firm and year, respectively; the dependent variable *OC*_*it*_ represents the manager of the firm, and i is overconfidence at t year; *Fintech*_*it*_ is measured by the digital financial inclusion index of firms’ registered location (prefecture-level city); and *CVs* refer to the control variables, which aim to control other heterogeneous characteristics at the firm level. This study controls for state-owned enterprise (*STATE*), firm size (*SIZE*), leverage ratio (*LEV*), firm age (*AGE*), board size (*BOARD*), Tobin Q (*Growth*), operating profit ratio (*GP*), the ratio of liquid assets relative to total assets (*AS*), and the shareholder ratio of the management team (*SR*). Furthermore, firm-specific fixed effects μ_i_ are added to the regression to account for time-invariant characteristics, and time-specific fixed effects are used to capture all time-variant macro-level factors that are common to firms. “A “ refers to the constant term. Specifically, to account for potential serial correlation and heteroscedasticity, we cluster standard errors at the industry level following the suggestion by Bertrand et al. (2004).

In Model (1), the coefficient of interest is β*_1_*, which indicates the effect of Fintech on manager overconfidence. β*_1_* should be significantly negative according to Hypothesis 1.

### 3.3 Manager overconfidence

Following the methodology proposed by Lin et al. (2005), this study constructs the measurement of manager overconfidence based on earning forecasts. Specifically, we define manager overconfidence as a dummy variable if the actual earnings are lower than the earnings forecasted by CEO or not for firm i in year t. *OC*_*it*_ is equal to 1 if the actual earnings are lower than the earnings forecasted by CEO or not for firm i in year t and is equal to 0 otherwise. Consistent with the methodology proposed by Lin et al. (2005), we hand-collected the earnings forecast data containing both mandatory forecasts and voluntary forecasts during 2011–2020 from firms’ announcements. The mandatory forecasts are required by the regulatory agency, China Securities Regulatory Commission (CSRC), and the voluntary forecasts are disclosed by firms through the media. This study follows Lin et al. (2005) and also assumes that the CEO has the final say in the team and that the forecasts are made by CEOs.

In addition, to further verify the robustness of our benchmark results, this study also follows Hayward and Hambrick (1997) and measures firms’ manager overconfidence each year by referring to the manager relative compensation method. The related processes are described as follows: (1) Calculating the ratio of the sum of the top three highest-paid executives’ compensation to the sum of all executives’ compensation; (2) calculating the ratio of the median of the top three highest-paid executives’ compensation to the median of all executives’ compensation; (3) manager overconfidence equals 1 if the ratio calculated by step (1) is larger than the ratio calculated by step (2) and 0 otherwise.

### 3.4 Measurements of Fintech: The digital financial inclusion index

With the rapid development of digital finance, the corresponding measurement standard is relatively thin. For a long time, digital finance development lacks accurate data to reflect its evolution trend. The lack of data measurement has led to some limitations in the research and observation of digital finance.

Under the efforts of the Chinese central and local governments, the Peking University Digital Financial Research Center and the Ant Financial Group Institute cooperation, “The Peking University Digital Financial Inclusion Index of China” has a strong representation, where the index to a certain extent alleviates the above situation, and enables all walks of life to understand China’s digital financial inclusion development status. The above database has been adopted to measure the development of Fintech in many studies (Bollaert et al., 2021; Lv and Xiong, 2022).

The general index of financial inclusion indicates the development status of digital inclusive digital finance in China. The three second-level indicators, respectively, represent the breadth of coverage, use depth, and digitalization degree of digital inclusive finance. The main source of the data is from the Alipay ecosystem and mainly describes the development level of digital inclusive finance in different regions, covering 31 mainland provinces, 377 cities above the prefecture level, and nearly 2,800 counties. The explanatory variable data selected in this study are from “The Peking University Digital Financial Inclusion Index of China (2011–2020),” released by Peking University.

### 3.5 Control variables

Following the previous related literature (Bollaert et al., 2021), this study defines the measurement of the other variables used as follows: State-owned enterprise (*STATE*), measured by a dummy variable equal to 1 if the firm’s ownership is stated-owned and 0 otherwise; firm size (*SIZE*) is measured by the natural logarithm of total assets; leverage (*LEV*) is the ratio of total liabilities divided by total assets; firm age (*AGE*) is measured by the number of years after the firm’s establishment; and *Growth* is measured by the Tobin Q. *GP* is measured by the Tobin Q as well as the operating profit ratio. *AS* is measured by the ratio of liquid assets relative to total assets. Board size (*BOARD*) is measured by the natural logarithm of the number of boards of directors of the firm; and the shareholder ratio of the management team (*SR*) is measured by the ratio of the shareholding of the largest shareholder.

### 3.6 Summary statistics

Table 2 presents the results of descriptive statistics for the main variables.

According to Table 2, the median value of manager overconfidence (*OC*) is 1 and the minimum value is 0. The median value of Fintech is 2.010, and the minimum and maximum values are 0.231 and 3.030, respectively, indicating that the overall situation of Fintech development is well, but there is still potential heterogeneity between different firm-registered locations. Therefore, the relationship between Fintech and manager overconfidence needs to be urgently investigated. In addition, the mean of the log of total assets (*SIZE*) is about 21.820, the mean of leverage ratio (*LEV*) is 0.390, and the standard deviation of Tobin Q (*Growth*) is relatively large, indicating a large difference in market values between the sample companies. The mean of the shareholder ratio of the management team (*SR*) is 0.167, which implies that the management team has the power to control the firm to some extent and plays a vital role in the firm’s decision-making. The mean of whether the firm is a state-owned enterprise or not (*STATE*) is 0.307, indicating that almost 30% of the sample firms are state-owned enterprises. The mean of the natural logarithm of years in the market (*AGE*) is 2.69, indicating that the average number of years in the market is about 8 years. The mean of the logarithm of board size (*BOARD*) is 2.131. The mean of the operating profit ratio (GP) is 0.296. In addition, all sample distributions are consistent with extant research.

Table 3 shows the correlation coefficients of the main variables. According to Table 3, it can be found that the correlation coefficients between the majority of variables are less than 0.5, indicating that the problem of multicollinearity between explanatory variables is not serious.

**TABLE 3:** Correlation coefficient matrix.

	*OC*	Fintech	*SR*	*LEV*	*GROWTH*	*GP*	*AS*	*SIZE*	*BOARD*	*AGE*	*STATE*
*OC*	1	0.052***	−0.058***	0.023***	–0.009	0.011	–0.008	0.056***	0.018**	–0.013	0.091***
Fintech	0.050***	1	0.138***	–0.005	0.072***	0.105***	0.006	0.118***	−0.141***	0.283***	−0.125***
*SR*	−0.053***	0.072***	1	−0.353***	0.315***	0.269***	0.230***	−0.342***	−0.195***	−0.253***	−0.564***
*LEV*	0.019**	−0.018**	−0.295***	1	−0.477***	−0.444***	−0.122***	0.544***	0.158***	0.201***	0.318***
*Growth*	0.008	0.080***	0.166***	−0.131***	1	0.378***	0.173***	−0.607***	−0.195***	−0.124***	−0.330***
*GP*	0.010	0.085***	0.225***	−0.379***	0.237***	1	0.097***	−0.208***	−0.088***	−0.073***	−0.229***
*AS*	–0.013	0.002	0.228***	−0.092***	0.111***	0.080***	1	−0.207***	−0.106***	−0.109***	−0.176***
*SIZE*	0.060***	0.113***	−0.346***	0.407***	−0.402***	−0.188***	−0.210***	1	0.236***	0.184***	0.357***
*BOARD*	0.033***	−0.136***	−0.197***	0.139***	−0.137***	−0.080***	−0.129***	0.255***	1	0.054***	0.268***
*AGE*	−0.013*	0.317***	−0.239***	0.170***	–0.004	−0.054***	−0.117***	0.140***	0.055***	1	0.201***
*STATE*	0.091***	−0.125***	−0.472***	0.274***	−0.171***	−0.212***	−0.203***	0.376***	0.277***	0.191***	1

This table reports the correlation coefficients between the variables, the lower triangular matrix reports the Pearson correlation coefficient, and the upper triangular matrix reports the Spearman correlation coefficient. The variable definitions are shown in Table 2. ***, **, and *represent the 1, 5, and 10% significance levels, respectively.

## 4 Results and discussion

### 4.1 Benchmark regression: Fintech and manager overconfidence

Table 4 provides the regression estimation results of Hypothesis 1. Column (1) in Table 4 shows the estimation results controlling for firm and year fixed effects, while column (2) shows the estimation results controlling for both firm, year fixed effects, individual and control variables.

**TABLE 4:** Benchmark regression results.

	*Dependent variable = OC*	
	Model (1)	Model (2)
Fintech	−0.077**	−0.077**
	(−2.14)	(−2.14)
*SR*		0.051***
		(8.32)
*LEV*		−0.018***
		(−13.21)
*Growth*		−0.118***
		(−8.89)
*GP*		–0.001
		(−0.85)
*AS*		−0.015***
		(−9.57)
*SIZE*		0.013**
		(2.13)
*BOARD*		0.126***
		(17.51)
*AGE*		0.007***
		(3.03)
*STATE*		–0.013
		(−0.64)
Constant	1.016***	0.934***
	(14.77)	(6.29)
Firm FE	Yes	Yes
Year FE	Yes	Yes
*N*	16,067	16,067
*R* ^2^	0.640	0.640
*F*	4.597	1.230

*t* statistic is in parenthesis. ***, **, and *represent the 1, 5, and 10% significance levels, respectively.

From the estimation results in column (1) of Table 4, the estimated coefficient of Fintech is significantly negative at a 5% level without control variables. The results in column (2) indicate that the estimated coefficient of Fintech remains significantly negative at a 5% level with control variables. All the above results support Hypothesis 1 that the development of Fintech decreases the level of manager overconfidence.

## 5 Robust check

### 5.1 Address potential endogenous problems: IV (instrumental variable) regression

Although this study has controlled variables correlated with manager overconfidence and Fintech development as much as possible, the robustness of our benchmark results is still threatened by potential endogeneity problems. The main reasons can be concluded as follow: (1) On one hand, Fintech development and manager overconfidence may have a reverse causal relationship. Lower levels of executive overconfidence may stimulate the demand for financial innovation tools to alleviate the operation stress faced by firms, for example, difficulty in financing, which objectively helps to promote the development of local Fintech. (2) On the other hand, some unobserved factors that change over time may still have an impact on the empirical results. Therefore, this study uses the average Fintech index of all other cities in the same province except the local city where the firm is located as the instrumental variable of the Fintech index of the firm’s location. The reasons for choosing this instrumental variable are (1) relevance. There is little difference in the resource support available to regions within the same province, and the level of Fintech development is similar. Moreover, due to the geographical proximity of regions within the same province, the level of Fintech development in other regions is likely to influence the Fintech development in the region to some extent. (2) Exogeneity: The level of Fintech development in other regions within the same province does not directly affect the level of manager overconfidence of firms in the region.

Table 5 shows the regression results using this instrumental variable, column (1) is the first-stage regression results, and the estimated coefficient of the instrumental variable is positive at a 1% significant level, indicating that the higher the level of Fintech development in other regions of the same province, the higher the level of Fintech in the location of the firm, indicating that the correlation between the instrumental variable selected in this study and the explanatory variable (Fintech) is strong, and the F-statistic of first-stage regression results is much larger than 10, indicating that the instrumental variable selected in this study is not weak. Column (2) shows the results of the second stage regression, and the estimated coefficient of Fintech is negative at the 5% significance level, indicating that after accounting for the endogenous issue, Fintech still has a significant negative effect on the manager overconfidence, which is consistent with our benchmark regression findings.

**TABLE 5:** Results of instrumental variables regression.

	Model (1)	Model (2)
	Fintech	*OC*
*IV*	0.964***	
	(115.37)	
Fintech		−0.121***
		(−2.38)
Constant	0.740***	1.202***
	(32.30)	(29.11)
Controls	Yes	Yes
Firm FE	Yes	Yes
Year FE	Yes	Yes
*N*	16,067	16,067
*R* ^2^	0.959	–0.002
*F*	238505.73	3.98

*t* statistic is in parenthesis. ***, **, and *represent the 1, 5, and 10% significance levels, respectively.

### 5.2 Alternative of manager overconfidence

To check the robustness of our benchmark results, we follow Hayward and Hambrick (1997) and measure manager overconfidence by referring to the manager relative compensation method. The related process is described as follows: (1) Calculating the ratio of the sum of the top three highest-paid executives’ compensation to the sum of all executives’ compensation; (2) calculating the ratio of the median of the top three highest-paid executives’ compensation to the median of all executives’ compensation; and (3) manager overconfidence equals 1 if the ratio calculated by step (1) is larger than the ratio calculated by step (2) and 0 otherwise.

The explanatory variables of model (1) and model (2) in Table 6 are the overconfidence variables calculated using the manager relative compensation method. By observing the regression results, the estimated coefficients of financial technology (Fintech) are all significantly negative at the 1% level.

**TABLE 6:** Robustness check.

	Alternative measurement of OC	Fintech_Lag
	Model (1)	Model (2)
Fintech	−0.0583***	
	(−5.63)	
Fintech _lag		−0.0248**
		(−2.11)
Constant	1.236***	0.970***
	(28.22)	(18.23)
Controls	Yes	Yes
Firm FE	Yes	Yes
Year FE	Yes	Yes
*N*	25,783	21,616
*R* ^2^	0.553	0.572
*F*	145.5	87.39

*t* statistic is in parenthesis. ***, **, and *represent the 1, 5, and 10% significance levels, respectively.

### 5.3 Other robustness checks

First, we use alternative explanatory variables to conduct a robustness check. Then, the Fintech variable in prefecture-level cities lagged by one period, and the estimation results are shown in model (3) of Table 6. The lagged one-period Fintech index (Fintech_Lag) is significantly negative at the 5% level.

Based on the above empirical results, it can be found that Fintech significantly reduces manager overconfidence after altering the measurement of manager overconfidence and lagging one-period Fintech variable for regression. Therefore, our benchmark results are robust.

In addition, because the measurement of manager overconfidence is a dummy variable, we employ the logic model, which serves as a classical discrete choice regression model, to test the effect of Fintech on manager overconfidence and reported related results in Appendix Table A1, which demonstrates the robustness of our main findings.

## 6 Heterogeneous analyses

The above empirical results have proved the relationship between Fintech and manager overconfidence. This section further examines the moderating effect of power concentration and financing constraints on the relationship between Fintech and manager overconfidence. In addition, this section also analyzes the channels through which Fintech affects manager overconfidence.

### 6.1 Power concentration

We followed several studies and defined power concentration as a dummy variable equal to 1 if the manager is also the chairman of the board and 0 if otherwise. Especially, power concentration is high if equal to 1 and otherwise. Then, we conduct sub-sample regression based on the power concentration. The results are shown in Table 7.

**TABLE 7:** Test the effect of power concentration.

	*Dependent variable = OC*	
	High power concentration	Low power concentration
	Model (1)	Model (2)
Fintech	–0.008	−0.103**
	(−1.08)	(−2.20)
Controls	Yes	Yes
Firm FE	Yes	Yes
Year FE	Yes	Yes
*N*	4,843	11,224
*R* ^2^	0.959	0.699
*F*	1.053	1.664

*t* statistic is in parenthesis. ***, **, and *represent the 1, 5, and 10% significance levels, respectively.

According to Table 7, we find that the negative effect of Fintech on manager overconfidence is only significant in the sample group with low power concentration.

### 6.2 Financing constraint

Based on Hadlock and Pierce (2010), the measurement method is quantified by using the absolute value to indicate the degree of financing constraint of the SA index. The greater the value is, the greater the corresponding financing constraint is. In this study, the sample is ranked by using the median log value of the absolute value of the SA index, dividing the samples into two groups of sample data with higher finance constraints (FC_High) and lower finance constraints (FC_Low).

As shown in Table 8, we find that the negative effect of Fintech on manager overconfidence is only significant in the sample group with high finance constraints.

**TABLE 8:** Test the effect of financing constraints.

	*Dependent variable = OC*	
	FC_high	FC_low
	Model (1)	Model (2)
Fintech	−0.131**	0.030
	(−2.41)	(0.61)
Controls	Yes	Yes
Firm FE	Yes	Yes
Year FE	Yes	Yes
*N*	8,033	8,034
*R* ^2^	0.693	0.644
*F*	1.783	1.672

*t* statistic is in parenthesis. ***, **, and *represent the 1, 5, and 10% significance levels, respectively.

### 6.3 The channels of Fintech affect manager overconfidence

The development of Fintech shows a diversified development trend, and the Peking University Inclusive Finance Index consists of three main dimensions, which are digital inclusive finance digitization degree, coverage breadth, and usage depth index. In this study, these three dimensions are used to measure the level of Fintech development in prefecture-level cities, respectively, and the estimation results are shown in Table 9. The results of model (3) indicate that the coefficient of the digitalization degree of financial digital inclusion is negative but not significant; the results of models (4) and (5) indicate that the estimated coefficients of the depth of coverage and depth of use of digital inclusion are significantly negative. Fintech reduces the degree of managerial overconfidence mainly by expanding the coverage of financial innovation services and increasing the depth of application of financial innovation tools, but the level of digital development of Fintech has an insignificant effect on the degree of managerial overconfidence.

**TABLE 9:** Channel analysis.

	*Dependent variable = OC*		
	Coverage breadth	Usage depth	Digitization level
	Model (1)	Model (2)	Model (3)
Fintech	0.001	−0.001***	−0.001**
	(1.39)	(−2.67)	(−2.38)
Constant	0.769***	0.930***	0.903***
	(5.61)	(6.49)	(6.38)
Controls	Yes	Yes	Yes
Firm FE	Yes	Yes	Yes
Year FE	Yes	Yes	Yes
*N*	16,067	16,067	16,067
*R* ^2^	0.640	0.640	0.640
*F*	0.968	1.487	1.342

*t* statistic is in parenthesis, ***, **, and *represent the 1, 5, and 10% significance levels, respectively.

## 7 Conclusion

Recently, Fintech in China experienced a boom in various aspects of daily life. The influence of Fintech on psychological outcomes began attracting the attention of many psychological scholars and economic scholars to investigate the impact of Fintech on manager overconfidence. Based on the panel data of Chinese A-share non-financial listed firms and the digital inclusive finance index of Chinese prefecture-level cities between 2011 and 2020, this study examines the relationship between Fintech and manager overconfidence. According to related empirical results, we find that the development of Fintech has a negative effect on manager overconfidence which helps to improve firms’ capacity for information acquisition and make more rational strategic decision-making for the firms. Based on the estimation result in Column (2) of Table 4, the estimated effects are positive, statistically significant, and economically meaningful. For instance, the results shown in Column (2) indicate that a one-standard-deviation increase in the Fintech level of a firm’s location decreases manager overconfidence by 2.681% points (this number is obtained by multiplying the standard deviation of Fintech by the estimated coefficient), which is consistent with those firms located in regions with higher levels of Fintech associated with a lower level of manager overconfidence. Furthermore, Xia et al. (2022) hold that firms located in regions with higher levels of Fintech experienced smaller losses during the COVID-19 pandemic and found that the coefficient on Fintech is significantly negative (coef. = 0.007, *t* = 4.791). Comparing previous literature about the effect of Fintech, this study’s coefficient on Fintech is significantly negative (coef. = –0.077, *t* = –2.14) and economically significant.

Moreover, the impact of Fintech on manager overconfidence varies across firm-specific characteristics containing financing constraints and power concentration. In addition, Fintech exerts a negative effect on manager overconfidence through channels containing Fintech coverage breadth and Fintech usage depth. Based on the above findings, this study provides relevant implications and recommendations as follows.

First, the firm itself needs to take the initiative when facing the impact brought by the development of Fintech. Firms should strengthen their sense of competition, steadily increase their investment in Fintech, enhance the ability to acquire information through internal research and external cooperation, actively empower firms’ development, improve enterprise managers’ ability, and help enterprises to develop sustainably. Second, the government should attach great importance to the role of Fintech, which is effective for long-term goals such as fostering the development of firms. Third, the government also needs to maintain the sustainability of the policy of Fintech. Moreover, the role of Fintech in firms with severe financing constraints and lower power concentration should be taken seriously. In addition, the government should increase the information and communication infrastructure, which could accelerate the digital transformation of firms and optimize the information environment of firms.

Finally, this study did not assess the impact of Fintech on manager overconfidence in different regions, and, in the future, we could consider various regional heterogeneities and’ re-examine the influence of Fintech on manager overconfidence.

## Data availability statement

The raw data supporting the conclusions of this article will be made available by the authors, without undue reservation.

## Author contributions

All authors listed have made a substantial, direct, and intellectual contribution to the work, and approved it for publication.

## Appendix

### Appendix A Other robustness check

**APPENDIX TABLE A1:** Logit model regression results.

	*Dependent variable* = *OC*	
	Model (1)	Model (2)
*Fintech*	–0.223***	–0.348***
	(–6.36)	(–8.81)
Constant	1.47***	–0.321
	(21.29)	(–0.52)
Controls	No	Yes
Firm FE	Yes	Yes
Year FE	Yes	Yes
*N*	16,067	16,067
seudo *R*^2^	0.003	0.023

*z* statistic is in parenthesis. ***, **, and *represent the 1, 5, and 10% significance levels, respectively.
